# 3D cone-beam CT with a twin robotic x-ray system in elbow imaging: comparison of image quality to high-resolution multidetector CT

**DOI:** 10.1186/s41747-020-00177-y

**Published:** 2020-09-08

**Authors:** Jan-Peter Grunz, Andreas Max Weng, Andreas Steven Kunz, Maike Veyhl-Wichmann, Rainer Schmitt, Carsten Herbert Gietzen, Lenhard Pennig, Stefan Herz, Süleyman Ergün, Thorsten Alexander Bley, Tobias Gassenmaier

**Affiliations:** 1 Department of Diagnostic and Interventional Radiology, University Hospital Würzburg, Oberdürrbacher Straße 6, 97080 Würzburg, Germany; 2 Institute of Anatomy and Cell Biology, University of Würzburg, Koellikerstraße 6, 97070 Würzburg, Germany; 3 Institute for Diagnostic and Interventional Radiology, Faculty of Medicine and University Hospital Cologne, University of Cologne, Kerpener Straße 62, 50937 Cologne, Germany

**Keywords:** Cancellous bone, Cone-beam computed tomography, Elbow, Elbow joint, Multidetector computed tomography

## Abstract

**Background:**

Elbow imaging is challenging with conventional multidetector computed tomography (MDCT), while cone-beam CT (CBCT) provides superior options. We compared intra-individually CBCT *versus* MDCT image quality in cadaveric elbows.

**Methods:**

A twin robotic x-ray system with new CBCT mode and a high-resolution clinical MDCT were compared in 16 cadaveric elbows. Both systems were operated with a dedicated low-dose (LD) protocol (equivalent volume CT dose index [CTDI_vol(16 cm)_] = 3.3 mGy) and a regular clinical scan dose (RD) protocol (CTDI_vol(16 cm)_ = 13.8 mGy). Image quality was evaluated by two radiologists (R1 and R2) on a seven-point Likert scale, and estimation of signal intensity in cancellous bone was conducted. Wilcoxon signed-rank tests and intraclass correlation coefficient (ICC) statistics were used.

**Results:**

The CBCT prototype provided superior subjective image quality compared to MDCT scans (for RD, *p* ≤ 0.004; for LD, *p* ≤ 0.001). Image quality was rated very good or excellent in 100% of the cases by both readers for RD CBCT, 100% (R1) and 93.8% (R2) for LD CBCT, 62.6% and 43.8% for RD MDCT, and 0.0% and 0.0% for LD MDCT. Single-measure ICC was 0.95 (95% confidence interval 0.91–0.97; *p* < 0.001). Software-based assessment supported subjective findings with less “undecided” pixels in CBCT than dose-equivalent MDCT (*p* < 0.001). No significant difference was found between LD CBCT and RD MDCT.

**Conclusions:**

In cadaveric elbow studies, the tested cone-beam CT prototype delivered superior image quality compared to high-end multidetector CT and showed a potential for considerable dose reduction.

## Key points

- The cone-beam computed tomography (CT) prototype provides better subjective image quality than dose-equivalent high-end multidetector CT in cadaveric elbow scans.
- Software-assisted differentiation between the cancellous bone and fatty marrow was superior using the new cone-beam CT scanning mode.
- Considerable dose reduction over multidetector CT is possible without loss of image quality.

## Background

Cone-beam computed tomography (CBCT) has been an integral part of dental imaging for many years [1, 2]. During the last decade, it has also made its way into musculoskeletal imaging [3].

With the excellent depiction of the osseous tissue and the establishment of dedicated extremity scanners, CBCT provides several advantages over conventional multidetector CT (MDCT), particularly for trauma imaging [4]. Current literature on CBCT is mostly focused on lower extremity and spine imaging under weight-bearing conditions [5–7]; however, upper extremity imaging benefits from CBCT as well.

While the upsides of the cone-beam technology have been examined for wrist and finger imaging before [8–10], the application to the elbow region has not been well established in the literature so far. Dislocations and fractures involving the elbow joint are common findings after trauma, especially in children [11]. The diagnostic routine usually comprises plain radiography for fracture diagnosis, although severely displaced fracture patterns or suspected occult coronoid or radial head fractures may require additional MDCT imaging for further evaluation and surgical planning [12].

Comparable to wrist scans, elbow imaging in MDCT is mostly performed in a modified so-called "superman" position to assure the best possible image quality and lowest radiation dose [13]. Yet, empirical knowledge shows that patients with elbow injuries or impaired shoulder movement and older patients in general are oftentimes unable to adopt or maintain that posture. Extremity-dedicated CBCT scanners provide more comfortable positioning options, allowing for optimal imaging conditions even if the patient has physical disabilities or is in pain [14]. However, due to technical limitations such as small field of view, many CBCT scanners can only be used for a limited number of imaging tasks, lacking the versatility of a conventional MDCT scanner [3].

The multifunctional x-ray device we employed in the study herein presented provides more flexibility. Its two telescopic arms and large detector allow for three-dimensional examination of most body parts and additional radiography and fluoroscopy. In earlier studies with a commercially available CBCT scan mode, a considerable reduction of radiation dose over standard MDCT was achieved for ankle [15] and wrist [16] studies using automatic tube current modulation. In this work, our goal was to establish reproducible clinical scan protocols with fixed acquisition parameters, thus enhancing comparability between the new CBCT prototype for the twin-robotic x-ray system and high-end MDCT in cadaveric elbow scans.

## Methods

### Cadaveric specimens

**Fig. 1:**
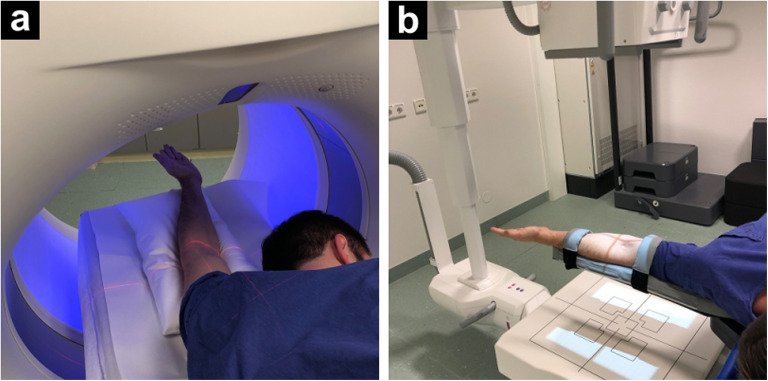
Positioning for image acquisition using a contemporary high-end multidetector computed tomography scanner (**a**) according to the modified “superman” position, and the twin robotic x-ray system cone-beam computed tomography scan mode (**b**) with the elbow positioned besides the patient table

We examined the elbow joints of eight, randomly chosen, formalin-fixed cadaveric specimens with the novel prototype cone-beam CT scan mode of a multi-use, twin robotic x-ray system (Multitom Rax, Siemens Healthineers, Erlangen, Germany) and a high-resolution multidetector CT scanner (Somatom Force, Siemens Healthineers, Erlangen, Germany). MDCT scans were conducted in a modified “superman” stance, while CBCT studies were performed using the tableside scan position for 3D imaging of the upper extremity. The scan postures for MDCT and CBCT elbow imaging are presented by a staff member in Fig. 1.

### Technical parameters and dose assessment

**Fig. 2:**
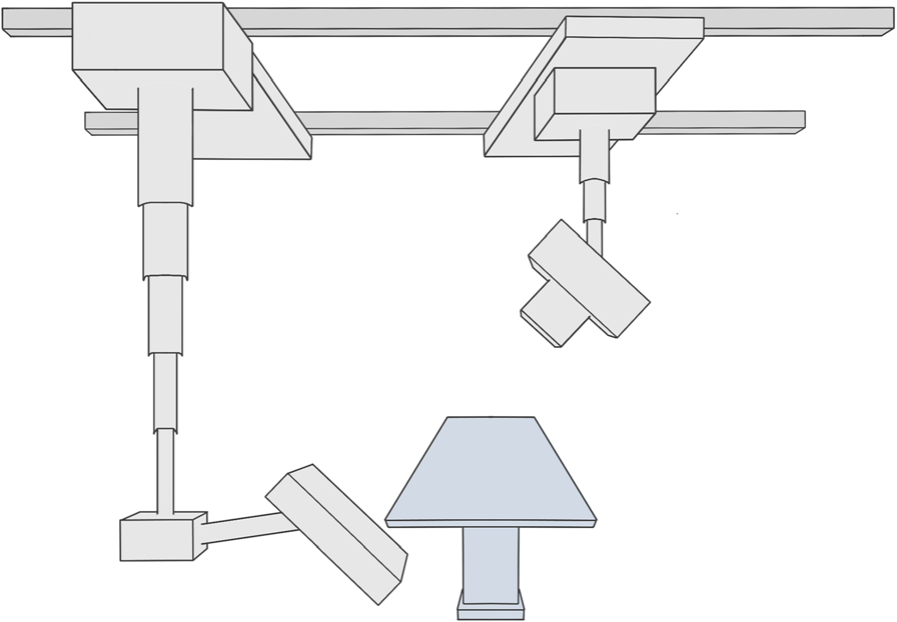
The prototype cone-beam computed tomography scan mode is based on a radiography system with two telescopic arms mounted on ceiling rails. One arm carries the x-ray tube, while the other holds a large flat panel detector. Simultaneous arm movement along predefined trajectories allows for three-dimensional projection data acquisition

The x-ray system tube and flat panel detector are mounted on two motor-driven, telescopic arms that are connected to ceiling rails. While independent arm movement facilitates two-dimensional and fluoroscopy imaging, both arms move concurrently along predetermined paths for the acquisition of 3D projection data in CBCT scan mode (Fig. 2). The input field of the flat panel detector measures 22.8 cm × 21.3 cm with a 3D image matrix of 1,540 × 1,440 pixels (pixel size 148 μm). The x-ray tube is capable of voltages from 40 to 150 kV and currents from 0.5 to 800 mAs.

The tableside trajectory used for elbow and wrist imaging has a sweep angle of 200° and an asymmetric source-to-image-distance of 115 cm. Due to an increase in maximum frames per second from 16 to 30, the total scan time is reduced with the new prototype from 20 to 12 s when compared to the commercially available software version. Taking into account the two telescopic arms acceleration and deceleration phase, 304 projection images are acquired during each scan. Dose levels and subsequently tube current can be varied for different scan protocols with an automatic dose modulation system keeping the detector dose level constant. Therefore, a sensor at the detector continuously measures the incoming radiation and the automatic exposure control modifies the tube current-time product accordingly to the preset dose values.

For this study, however, we decided to use constant tube currents to provide comparable circumstances for both imaging modalities. To compare the applied radiation dose between CBCT and MDCT, the dose-area product of the CBCT scan mode was multiplied by a linear scaling factor leading to volume computed tomography dose index (CTDI_vol_)-equivalent values. By using a polymethyl methacrylate dosimetry phantom, conforming to International Electrotechnical Commission 60601-2-44:2009 with a total length of 300 mm and a diameter of 160 mm as well as a standard dosimetry system (Nomex Dosimeter, PTW, Freiburg, Germany) with a 300 mm ionisation chamber, the scaling factor for each combination of voltage, pre-filtration and acquisition geometry was calculated in advance. To achieve this, the dose-length product (DLP) was quantified in five chambers. Then, the standard weighting scheme for dose measurements was applied to each value to determine the DLP_vol_ values. CTDI_vol_ values were calculated by dividing the DLP_vol_ by the beam width (equivalent to the field of view in the *z*-direction), and to finally compute the scaling factor, CTDI_vol_ was divided by dose-area product values. We operated with a dedicated low-dose (LD), *i.e.,* CTDI_vol(16 cm)_ = 3.3 mGy, and a regular dose protocol (RD) comparable to our clinical scan protocol, *i.e.,* CTDI_vol(16 cm)_ = 13.8 mGy), for both systems. MDCT images were acquired using the CT scanner in single-energy mode. Scan parameters for CBCT and MDCT studies are displayed in Table 1.

**Table 1:** Scan parameters

**Somatom Force**^**1**^	**regular-dose MDCT**	**low-dose MDCT**
Reference kVp	120	120
Reference mAs	100	25
CTDI_vol(16cm)_ (mGy)	13.8	3.3
Scan duration (s)	1	1
Collimation (mm)	2 × 32 × 0.3	2 × 32 × 0.3
Pitch (mm)	0.8	0.8
**Multitom Rax**^**1**^	**regular-dose CBCT**	**low-dose CBCT**
Reference kVp	80	80
Total scan mAs	757	210
CTDI_vol(16cm)_ (mGy)	13.8	3.3
Scan duration (s)	12	12
Max. frames per second	30	30
Projections per scan	304	304

***CTDI***_***vol(16cm)***_ *= volume computed tomography dose index (for 16 cm diameter PMMA dosimetry phantom)*

^*1*^*(Siemens Healthineers; Erlangen, Germany)*

Acquisition parameters for the regular-dose and dedicated low-dose scan protocols in prototype cone-beam CT (CBCT) and high-end multidetector CT (MDCT)

### Image reconstruction parameters

For the multidetector CT system, scanner-side reconstruction of raw data was conducted using a dedicated bone kernel (Ur77; Siemens Healthineers, Erlangen, Germany). The CBCT data was reconstructed with a comparable prototype kernel that provides equivalent standardised resolution numbers in the axial plane according to vendor information. Multiplanar reconstructions (MPR) of the cadaveric elbow scans were then performed for CBCT and MDCT using special software (Syngo via, Siemens Healthineers, Erlangen, Germany). Irrespective of dose protocol or scanner, slice thickness of 1 mm, increment of 0.5 mm, image matrix of 1,024 × 1,024 pixels and field of view of 80 mm were chosen for axial, coronal and sagittal planes. We decided on preset values for window width and level of 3,000 and 1,000 Hounsfield units for optimal bone visualisation. However, readers were allowed to change window settings as needed.

### Subjective image analysis

Two independent radiologists with nine (T.G.; Reader 1, R1) and seven (S.H.; Reader 2, R2) years of experience in musculoskeletal radiology evaluated all elbow studies using the Merlin picture archiving and communication system (Phönix-PACS, Freiburg, Germany). In the first step of their reads, observers reviewed all of the images for each MPR in randomised and blinded order. After blinded review of all data was complete, readers were asked to assess whether the presented image quality of each study was sufficient for diagnostic use and to rate the overall image quality on a seven-point Likert scale (7 = excellent; 6 = very good; 5 = good; 4 = satisfactory; 3 = fair; 2 = poor; 1 = very poor). Furthermore, artefacts and image noise in osseous and soft tissue were judged separately using a five-point Likert scale (5 = minimal artefacts or noise; 4 = little artefacts or noise; 3 = moderate artefacts or noise; 2 = considerable artefacts or noise; 1 = strong artefacts or noise).

### Objective image analysis

Software-based estimation of the signal intensity distribution in osseous tissue was performed in order to objectify the task of image quality evaluation. First, the range of signal intensities was calculated for image parts containing bone tissue. Subsequently, the fraction of pixels with a signal intensity between 25 and 75% of the calculated range was computed. Assuming that pixels above the 75%-border equal trabeculae or cortical bone and pixels below the 25%-border represent fatty marrow, the pixels between the borders were deemed as “undecided”, representing a mixture of bone and marrow and partly arising from blurred edges between both. Consequently, a larger share of these pixels indicated impaired image quality. We used special numerical computing software (MATLAB Version 2019a, MathWorks, Natick, MA, USA) for objective evaluation of image quality.

### Statistical analysis

Dedicated software (SPSS Statistics, Version 23.0 for Mac, IBM, Amonk, NY, USA) was used to carry out statistical analyses. We present categorical variables (*e.g.,* scale results) as frequencies, percentages and medians, whereas normally distributed data is presented as means ± standard deviation. The normal distribution of continuous variables was assessed using Kolmogorov-Smirnov tests. Paired nonparametric variables were compared using Wilcoxon signed-rank tests. For quantification of interrater reliability, we report the intraclass correlation coefficient (ICC) based on the absolute agreement in a two-way random-effects model. The interpretation of ICC values was conducted according to Koo and Li [17] (< 0.50 = poor reliability; 0.50–0.75 = moderate reliability; 0.75–0.90 = good reliability; > 0.90 = excellent reliability). The *p* values ≤ 0.05 were considered to indicate statistical significance.

## Results

### Subjective image quality

**Fig. 3:**
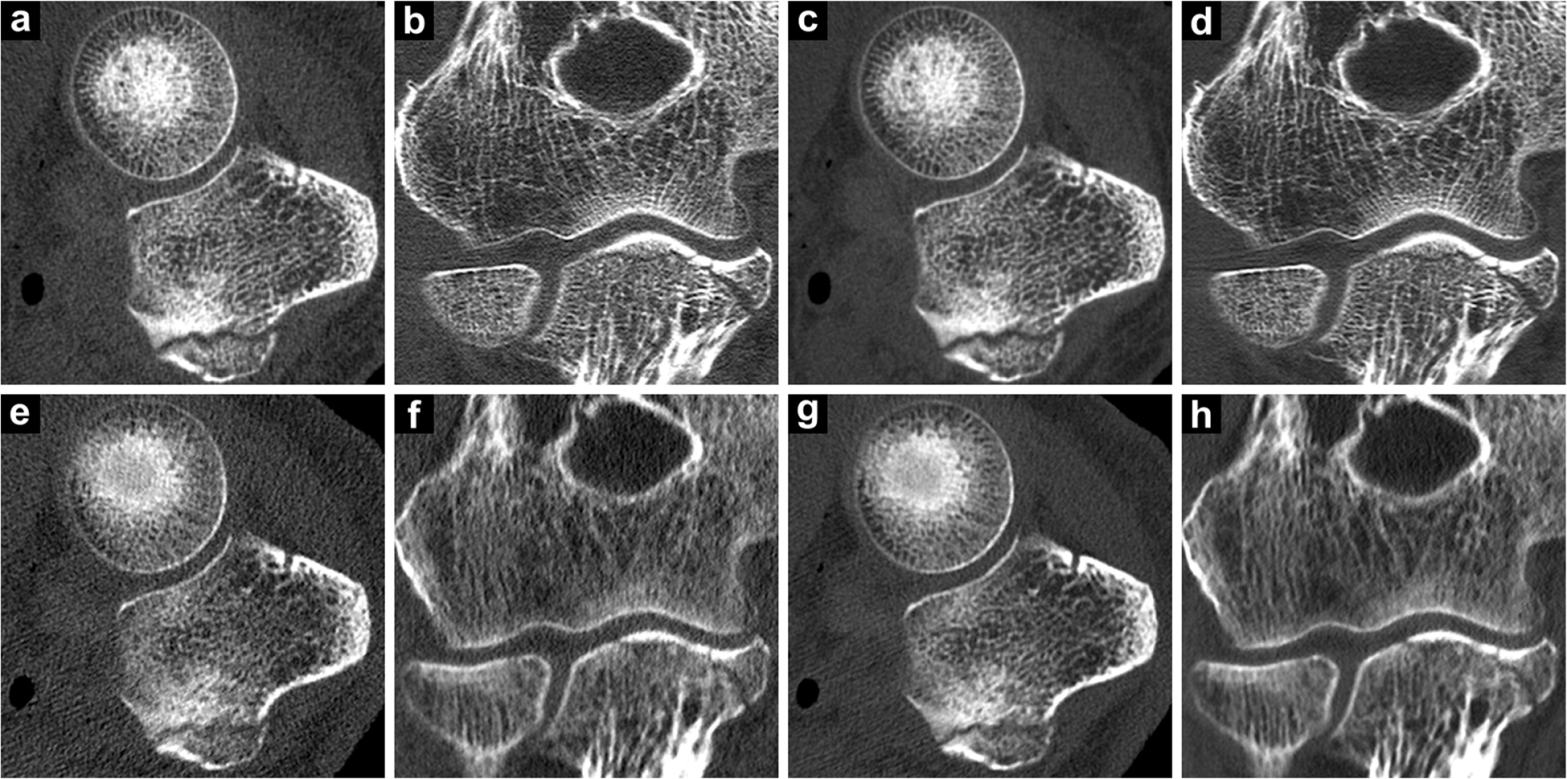
Representative cone-beam computed tomography (CBCT) and multidetector computed tomography (MDCT) images in the axial and coronal plane with equivalent low-dose and regular-dose scan protocols for visualisation of image quality in cadaveric elbow scans. Images depict an olecranon fracture as an incidental finding. **a**, **b** Low-dose CBCT. **c**, **d** Regular-dose CBCT. **e**, **f** Low-dose MDCT. **g**, **h** Regular-dose MDCT

Of 64 elbow studies, both readers considered all 16 RD CBCT, 16 LD CBCT, and 16 RD MDCT scans to be suitable for clinical evaluation. For LD MDCT examinations, R1 deemed 37.5% of studies and R2 31.3% of studies insufficient for diagnostic use (score 1 or 2). In contrast, R1 and R2 found the overall image quality to be very good or excellent (score 6 or 7) in 100% and 100% of RD CBCT studies, in 100% and 93.8% of LD CBCT studies, in 62.5% and 43.8% of RD MDCT studies, and in 0.0% and 0.0% of LD MDCT studies, respectively. Median Likert scores for R1 and R2 were 7.0 and 7.0 for RD CBCT studies, 6.0 and 6.5 for LD CBCT studies, 6.0 and 5.0 for RD MDCT studies and 3.5 and 3.0 for LD MDCT studies. Significantly superior image quality of CBCT studies in comparison to dose-equivalent MDCT studies was assessed by both readers: RD protocols, *p* ≤ 0.002; LD protocols, *p* ≤ 0.001. Low-dose CBCT studies were also rated preferably over RD MDCT by both readers (*p* ≤ 0.004). Figure 3 depicts representative coronal and axial MPRs of CBCT and MDCT scans for illustration of overall image quality. Single-measure ICC for overall image quality was 0.95 (95% confidence interval 0.91–0.97, *p* < 0.001). Table 2 shows detailed results of subjective image quality ratings by both radiologists.

**Table 2:** Subjective evaluation of cone-beam (CBCT) and multidetector CT (MDCT) image quality by two radiologists (R1, R2) using a seven-point Likert scale

	Score	Regular-dose CBCT		Low-dose CBCT		Regular-dose MDCT		Low-dose MDCT	
		R1	R2	R1	R2	R1	R2	R1	R2
Overall image quality	7	16 (100.0)	13 (81.3)	7 (43.8)	8 (50.0)	1 (6.3)	3 (18.8)	–	–
	6	–	3 (18.8)	9 (56.3)	7 (43.8)	9 (56.3)	4 (25.0)	–	–
	5	–	–	–	1 (6.3)	–	3 (18.8)	2 (12.5)	5 (31.3)
	4	–	–	–	–	4 (25.0)	3 (18.8)	6 (37.5)	2 (12.5)
	3	–	–	–	–	2 (12.5)	3 (18.8)	2 (12.5)	4 (25.0)
	2	–	–	–	–	–	–	5 (31.3)	3 (18.8)
	1	–	–	–	–	–	–	1 (6.3)	2 (12.5)
	**Median**	**7.0**	**7.0**	**6.0**	**6.5**	**6.0**	**5.0**	**3.5**	**3.0**

Image quality ordinal score: 7 = excellent; 6 = very good; 5 = good; 4 = satisfactory; 3 = fair; 2 = poor; 1 = very poor. Scale results are displayed as frequencies (percentages) and median values

Both observers rated image noise in the bone as less prominent in CBCT than MDCT scans (RD, *p* ≤ 0.023; LD, *p* ≤ 0.007). Noise in the soft tissue was unanimously conceived minor in CBCT for the LD protocol (*p* ≤ 0.006), while R2 also found less soft tissue noise for the RD CBCT protocol (*p* = 0.019). For artefacts in osseous tissue, no difference was assessed between CBCT and MDCT. In the soft tissue, artefacts were continuously more distinct in CBCT than MDCT scans for both readers (RD, *p* ≤ 0.003; LD, *p* ≤ 0.002) (Table 3).

**Table 3:** Assessment of image noise and artefacts in the bone and soft tissue for cone-beam (CBCT) and multidetector CT (MDCT) by two radiologists (R1, R2) using a five-point Likert scale

	Score	Regular-dose CBCT		Low-dose CBCT		Regular-dose MDCT		Low-dose MDCT	
		R1	R2	R1	R2	R1	R2	R1	R2
Noise in the bone	5	16 (100.0)	14 (87.5)	–	6 (37.5)	10 (62.5)	2 (12.5)	–	–
	4	–	2 (12.5)	10 (62.5)	7 (43.8)	4 (25.0)	8 (50.0)	6 (37.5)	4 (25.0)
	3	–	–	6 (37.5)	3 (18.8)	2 (12.5)	4 (25.0)	3 (18.8)	4 (25.0)
	2	–	–	–	–	–	2 (12.5)	3 (18.8)	4 (25.0)
	1	–	–	–	–	–	-	4 (25.0)	4 (25.0)
	**Median**	**5.0**	**5.0**	**4.0**	**4.0**	**5.0**	**4.0**	**3.0**	**2.5**
Noise in the soft tissue	5	1 (6.3)	–	–	–	2 (12.5)	2 (12.5)	–	–
	4	10 (62.5)	14 (87.5)	–	–	6 (37.5)	2 (12.5)	–	–
	3	5 (31.3)	2 (12.5)	7 (43.8)	9 (56.3)	8 (50.0)	10 (62.5)	2 (12.5)	2 (12.5)
	2	–	–	5 (31.3)	4 (25.0)	–	2 (12.5)	2 (12.5)	2 (12.5)
	1	–	–	4 (25.0)	3 (18.8)	–	–	12 (75.0)	12 (75.0)
	**Median**	**4.0**	**4.0**	**2.0**	**3.0**	**3.5**	**3.0**	**1.0**	**1.0**
Artefacts in the bone	5	16 (100.0)	16 (100.0)	16 (100.0)	15 (93.8)	13 (81.3)	12 (75.0)	13 (81.3)	12 (75.0)
	4	–	–	–	1 (6.3)	2 (12.5)	4 (25.0)	2 (12.5)	2 (12.5)
	3	–	–	–	–	–	–	1 (6.3)	2 (12.5)
	2	–	–	–	–	–	–	–	–
	1	–	–	–	–	–	–	–	–
	**Median**	**5.0**	**5.0**	**5.0**	**5.0**	**5.0**	**5.0**	**5.0**	**5.0**
Artefacts in the soft tissue	5	–	–	–	–	13 (81.3)	12 (75.0)	13 (81.3)	12 (75.0)
	4	15 (93.8)	12 (75.0)	15 (93.8)	7 (43.8)	2 (12.5)	3 (18.8)	2 (12.5)	2 (12.5)
	3	1 (6.3)	2 (12.5)	1 (6.3)	5 (31.3)	1 (6.3)	1 (6.3)	1 (6.3)	2 (12.5)
	2	–	2 (12.5)	–	4 (25.0)	–	–	–	–
	1	–	–	–	–	–	–	–	–
	**Median**	**4.0**	**4.0**	**4.0**	**3.0**	**5.0**	**5.0**	**5.0**	**5.0**

Image quality ordinal score: 5 = minimal artefacts or noise; 4 = little artefacts or noise; 3 = moderate artefacts or noise; 2 = considerable artefacts or noise; 1 = strong artefacts or noise). Scale results are displayed as frequencies (percentages) and median values

When comparing both CBCT scan protocols for the elbow imaging on the twin robotic x-ray system, both readers conceived RD CBCT to be superior to LD CBCT for image noise in the bone (*p* ≤ 0.005) and soft tissue (*p* ≤ 0.001). Regarding artefacts, R2 found less of them in soft tissue for RD studies (*p* = 0.008), while no significant differences were observed for artefacts in osseous tissue (*p* ≥ 0.317).

### Objective image quality

**Fig. 4:**
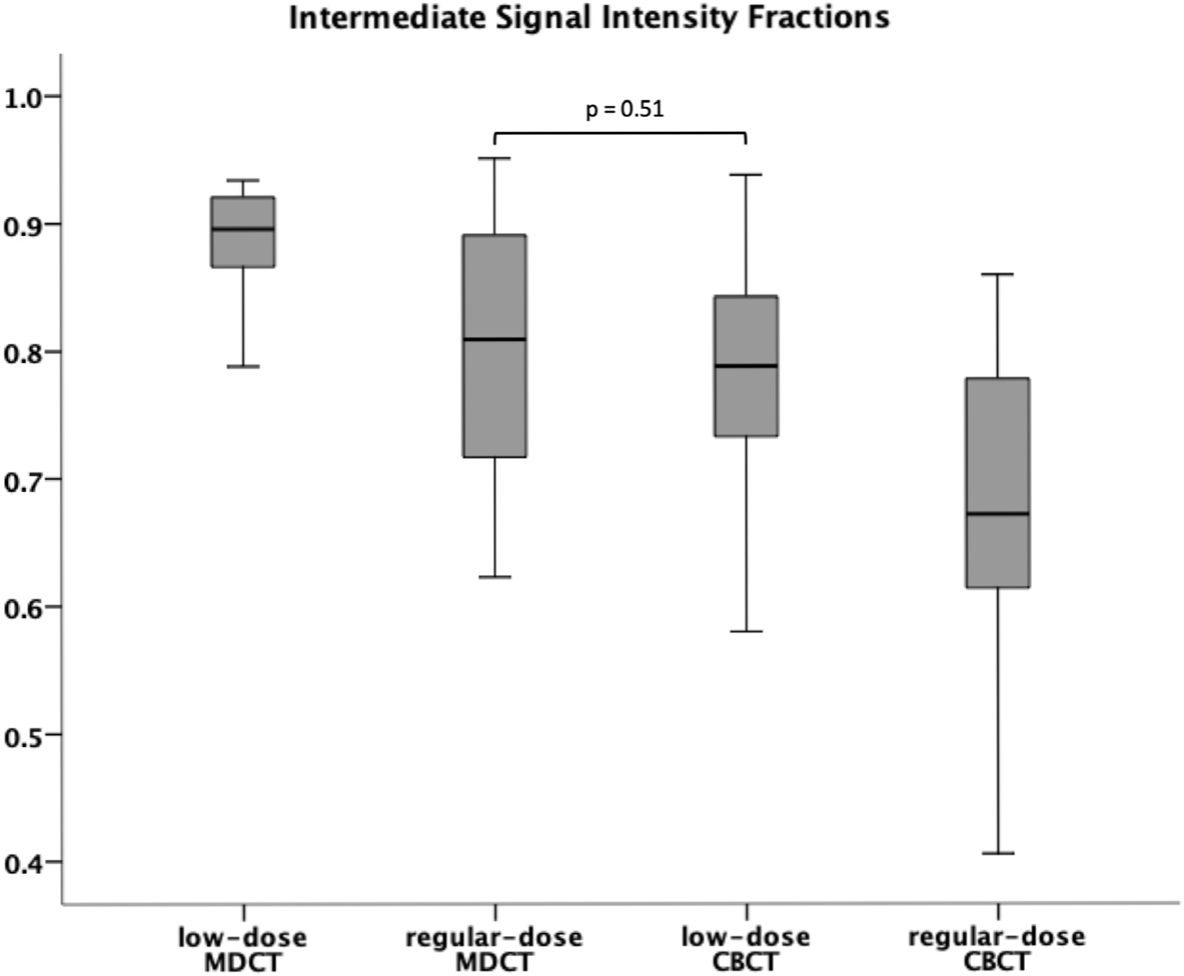
Boxplots (median and 50% of cases within the boxes) illustrate “undecided” pixel fractions within the intermediate range (25–75%) of signal intensities for cone-beam computed tomography (CBCT) and multidetector computed tomography (MDCT) scans with dose-equivalent scan protocols. Smaller pixel fractions with intermediate signal intensity indicate superior image quality. Except for the difference between regular-dose MDCT and low-dose CBCT, all differences are statistically significant (*p* < 0.001)

**Fig. 5:**
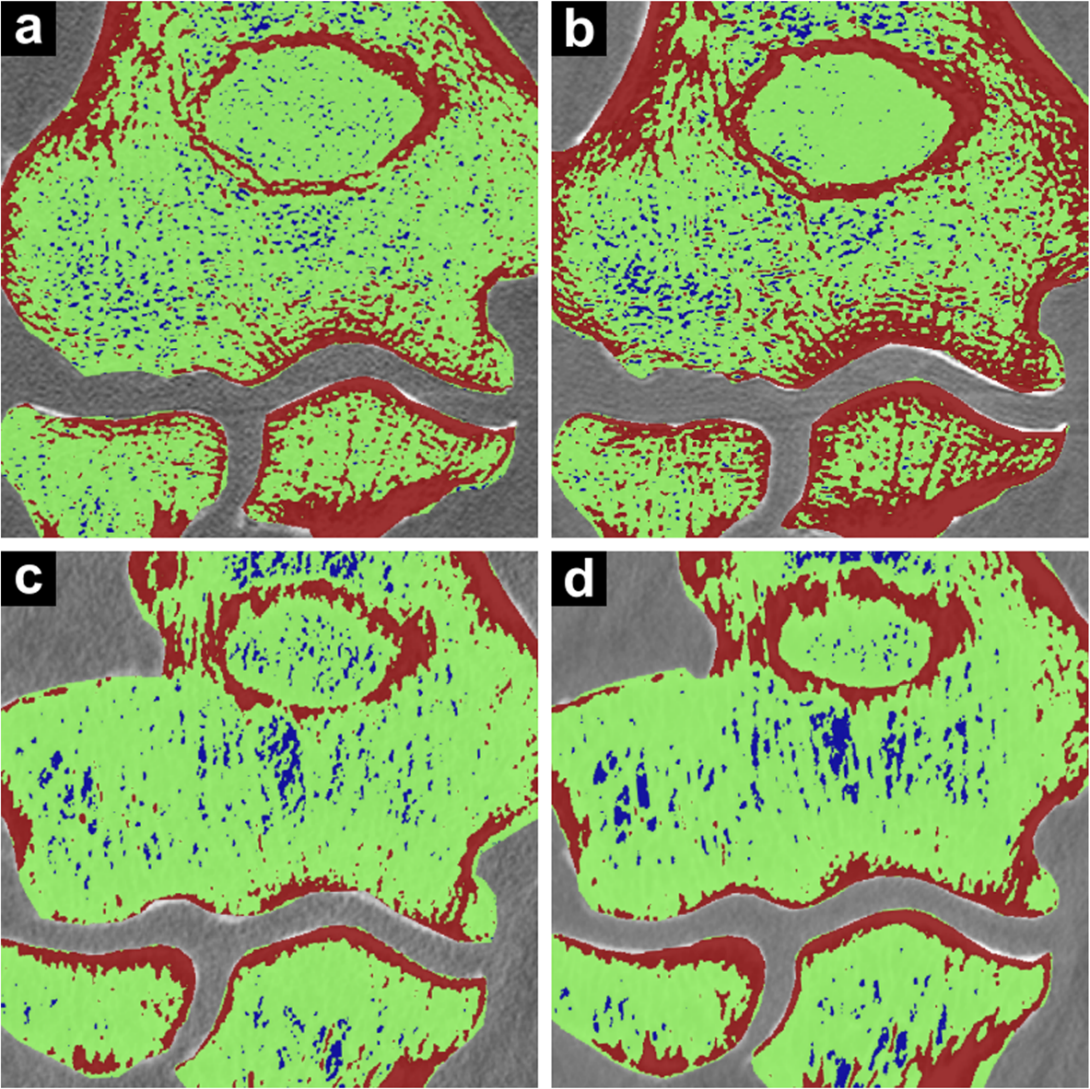
Colour-coded visualisation of signal intensity distribution in cancellous bone for cone-beam computed tomography (CBCT) and multidetector computed tomography (MDCT) scans. Red regions contain the maximum quartile of grey values (75–100%), while blue regions represent the minimum quartile (0–25%). Green regions consist of pixels with intermediate signal intensity (25–75%). **a** Low-dose CBCT. **b** Regular-dose CBCT. **c** Low-dose MDCT. **d** Regular-dose MDCT

As displayed in Fig. 4, RD CBCT (median fraction 0.67, IQR 0.16) delivered a smaller fraction of pixels within the 25% to 75% range of signal intensity than LD CBCT (0.79, IQR 0.08; *p* < 0.001), RD MDCT (0.81, IQR 0.17; *p* < 0.001), and LD MDCT scans (0.90, IQR 0.05; *p* < 0.001). While LD CBCT scans provided less “undecided” pixels than dose-equivalent LD MDCT scans (*p* < 0.001), no significant difference was found between LD CBCT and RD MDCT studies (*p* = 0.513). The signal intensity distribution in cancellous bone is visualised in Fig. 5.

## Discussion

We compared the image quality of a multi-use x-ray system prototype CBCT scan mode to a high-end MDCT using CTDI_vol_-matched, clinical RD and dedicated LD protocols for cadaveric elbow imaging on both scanners. Two radiologists rated CBCT scans to feature superior overall image quality compared to dose-equivalent MDCT studies. Reliability proved to be excellent between observers. This was further supported by the superior results of CBCT scans in objective image quality analysis of cancellous bone. The best distinction of the trabecula and bone marrow was achieved with the regular-dose scan protocol of the CBCT prototype. Despite four times lower radiation dose through tube current reduction, the computer-assisted evaluation of signal intensity distribution in the osseous tissue found no substantial difference between LD CBCT and RD MDCT studies. Observers even stated that LD CBCT studies featured better overall image quality (analysis of bone and soft tissue) than RD MDCT studies. All LD CBCT studies were deemed diagnostic, whereas the application of the LD MDCT protocol resulted in approximately one third non-diagnostic images.

The superior image quality of the CBCT prototype can most likely be attributed to the twin robotic x-ray system detector providing an isotropic voxel size of 149 μm, while the contemporary MDCT scanner has a pixel size of 300 μm in the axial plane and 600 μm in the *z*-direction. Furthermore, the limiting effect of the focal spot size on spatial resolution is reduced in CBCT scans by using a low-magnification acquisition geometry. In contrast to a previous ankle study with the commercially available CBCT scan mode, the tested prototype did not display more artefacts in bone than MDCT [15]. However, consistent with the literature [18], typical cone-beam artefacts were still present in the soft tissue. While cone-beam scans were at least equal to MDCT concerning soft tissue noise, image noise in the bone was less pronounced for CBCT. The excellent depiction of bone microarchitecture and minimised radiation dose in CBCT scans has been one of the main reasons for its prominent role in oral and maxillo-facial imaging [19, 20]. However, unlike other CBCT scanners, the prototype 3D scan mode of the multi-use x-ray system does not suffer from below-average image quality in the soft tissue [21]. Although trauma imaging of the elbow joint is primarily focused on bone display for fracture detection, the higher soft-tissue contrast can also be important, for example in occult fractures with concomitant effusion.

Regarding positioning options, the two telescopic arms and tableside scan trajectory of the twin robotic x-ray system enable elbow scans in a comfortable supine or seated position with the upper extremity abducted by 90°. As patients with elbow trauma are frequently unable to adopt or maintain the challenging scan position necessary for optimal multidetector CT elbow studies, they may still be examined using the CBCT scan mode without higher radiation dose or loss of image quality.

Furthermore, the availability of radiography, fluoroscopy and CBCT imaging within the x-ray suite allows for potential one-stop-shop imaging after trauma without additional patient transport or repeated positioning. Taking into account that elbow injuries (such as supracondylar humerus fracture) oftentimes affect children, the combination of first-rate 3D image quality and low radiation dose might particularly find application in paediatric radiology.

Limiting this study, 16 elbow joints from eight body donors were evaluated. As scans were exclusively performed on cadaveric specimens, possible motion artefacts cannot be ruled out for patient studies [3, 22]. The tested software prototype already reduced total scan time from 20 to 12 s compared to the current commercially available scan mode. However, this might still result in more artefacts through movement in clinical studies. The first tests of a marker-free auto-focus method based on the grey-level histogram entropy suggest that the acquisition time of the CBCT mode could be reduced even further by compensating deviations from the assumed scanning trajectory [23]. This approach should be evaluated in subsequent patient studies. As the tested CBCT prototype does not feature the same calibration for Hounsfield units as the MDCT scanner, signal-to-noise and contrast-to-noise ratios could not be compared in this study, subsequently limiting the availability of quantitative data. Furthermore, the impact of metallic implants or a cast around the elbow could not be analysed, because no osteosynthesis material was present in the field of view for any specimen. Formalin fixation is known to affect biological tissue samples over time, *e.g.,* by breaking of hydrogen bonds and protein denaturation [24]. With specimens embalmed in formalin for several years before this study, optical density might have decreased, irrespective of imaging modality [25]. While Seidel et al. [26] found no substantial difference between the diagnostic accuracy of MDCT and CBCT scans of a cadaveric specimen with and without formalin fixation, particularly the long-term effects of such fixation are not fully understood. Moreover, despite the blinded evaluation of scans, readers might have become accustomed to the characteristic image traits of each scanner over the time of their reads.

Finally, we note that the presented CBCT prototype is currently not commercially available. Siemens Healthineers Multitom Rax is not available in all countries. Due to regulatory reasons, its future availability cannot be guaranteed.

In conclusion, the tested CBCT scan mode of the twin robotic x-ray system provided better image quality than high-resolution MDCT for regular and low-dose scans of cadaveric elbows. The dedicated low-dose CBCT scan protocol delivered equally good distinction of bone microarchitecture and marrow as regular clinical MDCT studies despite one fourth of radiation dose, thus offering the potential for dose reduction.

## Data Availability

The datasets used and/or analysed during the current study are available from the corresponding author on reasonable request.

## Abbreviations

3D: Three-dimensional
CBCT: Cone-beam computed tomography
CTDI_vol(16 cm)_: Volume computed tomography dose index (for 16-cm diameter polymethyl methacrylate dosimetry phantom)
DLP: Dose-length product
ICC: Intraclass correlation coefficient
LD: Low-dose scan protocol (CTDI_vol(16 cm)_ = 3.3 mGy)
MPR: Multiplanar reconstruction
R1: Reader 1
R2: Reader2
RD: Regular dose (clinical) scan protocol (CTDI_vol(16 cm)_ = 13.8 mGy)
